# Yeast 9-1-1 complex acts as a sliding clamp for DNA synthesis by DNA polymerase ε

**DOI:** 10.1016/j.jbc.2022.102727

**Published:** 2022-11-19

**Authors:** Narottam Acharya, Louise Prakash, Satya Prakash

**Affiliations:** Department of Biochemistry and Molecular Biology, University of Texas Medical Branch, Galveston, Texas, USA

**Keywords:** DNA Polε, PCNA, 9-1-1 clamp, yeast 17-3-1 clamp, 17-3-1-dependent DNA synthesis by Polε, BER, base excision repair, GST, glutathione-*S*-transferase, IDCL, interdomain connector loop, PCNA, proliferating cell nuclear antigen, PIP, PCNA-interacting peptide, Polδ, DNA polymerase δ, Pol2, DNA polymerase 2, RFC, replication factor C, RPA, replication protein A

## Abstract

Eukaryotic cells harbor two DNA-binding clamps, proliferating cell nuclear antigen (PCNA), and another clamp commonly referred to as 9-1-1 clamp. In contrast to the essential role of PCNA in DNA replication as a sliding clamp for DNA polymerase (Pol) δ, no such role in DNA synthesis has been identified for the human 9-1-1 clamp or the orthologous yeast 17-3-1 clamp. The only role identified for either the 9-1-1 or 17-3-1 clamp is in the recruitment of signal transduction kinases, which affect the activation of cell cycle checkpoints in response to DNA damage. However, unlike the loading of PCNA by the replication factor C (RFC) clamp loader onto 3′-recessed DNA junctions for processive DNA synthesis by Polδ, the 17-3-1 clamp or the 9-1-1 clamp is loaded by their respective clamp loader Rad24-RFC or RAD17-RFC onto the 5′-recessed DNA junction of replication protein A–coated DNA for the recruitment of signal transduction kinases. Here, we identify a novel role of 17-3-1 clamp as a sliding clamp for DNA synthesis by Polε. We provide evidence that similar to the loading of PCNA by RFC, the 17-3-1 clamp is loaded by the Rad24-RFC clamp loader at the 3′-recessed DNA junction in an ATP-dependent manner. However, unlike PCNA, the 17-3-1 clamp does not enhance the processivity of DNA synthesis by Polε; instead, it greatly increases the catalytic efficiency of Polε for correct nucleotide incorporation. Furthermore, we show that the same PCNA-interacting peptide domain in the polymerase 2 catalytic subunit mediates Polε interaction with the 17-3-1 clamp and with PCNA.

Although proliferating cell nuclear antigen (PCNA) and the 9-1-1 clamp share a similar ring structure and are loaded onto DNA, they differ in many aspects and function in different cellular processes. PCNA is a homotrimer that is loaded onto DNA by the replication factor C (RFC) clamp loader, whereas the heterotrimer human 9-1-1 clamp comprised of RAD9, HUS1, and RAD1, is loaded by RAD17-RFC (1, 2). The orthologous yeast 17-3-1 clamp is comprised of the Rad17, Mec3, and Ddc1 and loaded onto DNA by Rad24-RFC (3). Both clamp loaders share the same four small Rfc2–5 subunits but differ in their largest subunit, which is Rfc1 in the canonical RFC, and Rad24 in Rad24-RFC (4). RFC and Rad24-RFC are specific in the loading of PCNA and 17-3-1 clamp, respectively. While RFC loads PCNA onto 3′-recessed DNA junctions, which greatly increases the processivity of replicative polymerase (Pol) δ (5, 6), Rad24-RFC loads the 17-3-1 clamp onto 5′-recessed DNA junctions of replication protein A (RPA)–coated DNA (7). A similar specificity is seen in the loading of human 9-1-1 clamp onto 5′-DNA junctions by the hRAD17-RFC (1). By contrast to the essential role of PCNA and its loader in DNA replication, the 17-3-1 clamp and Rad24 are not essential for survival, and they play no direct role in replication.

All the available information thus far has indicated a role of human 9-1-1 and yeast 17-3-1 clamp in the recruitment of signal transduction kinases onto 5′-recessed DNA junctions that form in response to DNA damage (8, 9, 10, 11, 12). Although the role of yeast 17-3-1 clamp and human 9-1-1 clamp in the activation of yMec1 and hATR kinase, respectively, *via* their loading onto 5′-recessed junctions of RPA-coated DNA to initiate DNA damage–induced checkpoint response has been documented extensively, there has been no information on whether the 17-3-1 or 9-1-1 clamp can activate DNA synthesis by a DNA polymerase, wherein the 17-3-1 (9-1-1) clamp is loaded onto 3′-recessed DNA junctions by Rad24-RFC (RAD17-RFC), akin to the loading of PCNA by RFC for DNA synthesis by Polδ. Here, we provide evidence for the role of 17-3-1 clamp that has been loaded onto the 3′-recessed DNA junction by Rad24-RFC, as a sliding clamp for DNA synthesis by Polε. Furthermore, we show that a single PCNA-interacting peptide (PIP) domain in the polymerase 2 (Pol2) catalytic subunit governs the functional interaction of Polε with both the 17-3-1 clamp and with PCNA.

## Results

### Stimulation of DNA synthetic activity of Polε by 17-3-1 clamp

**Figure 1:**
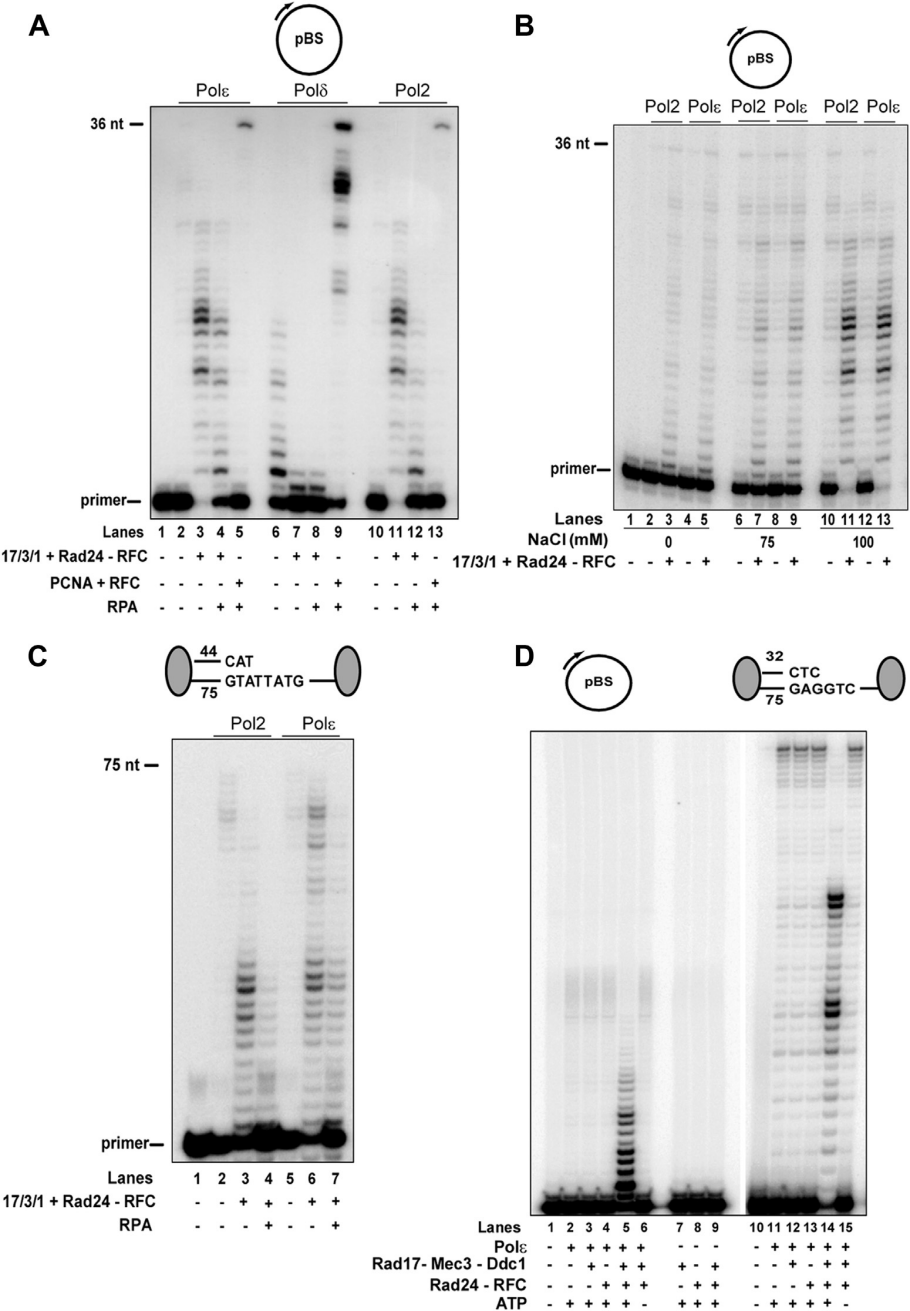
**Effect of 17-3-1 clamp on DNA synthesis by Polε.***A*, the 17-3-1 clamp stimulates DNA synthesis by Polε. 0.5 nM each of purified Polε (lanes 2–5), Polδ (lanes 6–9), or the Pol2 catalytic subunit of Polε (lanes 10–13) was incubated with the circular ssDNA substrate (5 nM) in the presence of a mixture of 100 μM dGTP, dCTP, and dATP under standard reaction conditions. As indicated, the reactions were carried out in the presence or the absence of 17-3-1 (40 ng) and Rad24-RFC (10 ng), or PCNA (100 ng) and RFC (25 ng), and with or without RPA (200 ng). Lane 1, DNA substrate alone. *B*, effect of salt on 17-3-1-dependent DNA synthesis by Pol2 and Polε. 0.5 nM of Pol2 or Polε was incubated with the circular ssDNA substrate (5 nM) under conditions as used in (*A*), except that the NaCl concentration was varied as indicated (0–100 mM). Lane 1, DNA substrate alone. *C*, effect of 17-3-1 clamp on DNA synthesis by Polε and Pol2 on linear DNA substrate. 0.5 nM of Pol2 (lanes 2–4) or Polε (lanes 5–7) was incubated with a biotin–streptavidin-bound linear DNA substrate (10 nM) in the presence of 100 μM each of four dNTPs under standard reaction conditions. As indicated, the reactions were carried out in the presence or the absence of 17-3-1 (40 ng) and Rad24-RFC (10 ng), with or without RPA (100 ng). Lane 1, DNA substrate alone. In this and other figures, the biotin–streptavidin complexes bound at the template ends are indicated by *dark ovals*. *D*, requirement of loading of 17-3-1 clamp by Rad24-RFC for DNA synthesis by Polε. 0.5 nM of Polε (lanes 2–6 and 11–15) was incubated with circular ssDNA substrate (10 nM) in the presence of 100 μM dGTP, dATP, and dCTP (lanes 2–6) or with linear DNA substrate (10 nM) with 100 μM each of the four dNTPs (lanes 11–15) under standard reaction conditions. As indicated, the reactions were carried out in the presence or the absence of 17-3-1 (20 ng), Rad24-RFC (5 ng), and ATP. In lanes 7 to 9, no DNA polymerase was added to the reaction and circular ssDNA substrate (10 nM) was used. Lanes 1 and 10, DNA substrate alone. PCNA, proliferating cell nuclear antigen; Pol2, DNA polymerase 2; Polε, DNA polymerase ε; RFC, replication factor C; RPA, replication protein A.

Since PCNA is loaded onto DNA by RFC specifically at the 3′-recessed junction and stimulates the processivity of Polδ, we examined whether the 17-3-1 clamp could also be loaded onto 3′-recessed DNA junctions by Rad24-RFC and affect DNA synthesis by Polδ or Polε. For these experiments, DNA synthesis was examined in the presence of 100 mM NaCl, which approximates the *in vivo* salt concentration. Although PCNA loaded by RFC-stimulated DNA synthesis by Polδ, (Fig. 1*A*, lane 9), we find that DNA synthesis by Polδ is inhibited upon the addition of 17-3-1 clamp and Rad24-RFC loader (Fig. 1*A*, lanes 6–8), as has been reported previously (7). In striking contrast, DNA synthesis by Polε was greatly stimulated in the presence of 17-3-1 clamp and Rad24-RFC loader (Fig. 1*A*, compare lanes 2 and 3). However, unlike the increased processivity of Polε imparted by PCNA (Fig. 1*A*, lane 5), 17-3-1 greatly increased primer usage, and DNA synthesis appeared distributive (Fig. 1*A*, lane 3). Since RPA is inhibitory to the loading of 17-3-1 clamp at 3′-recessed DNA junctions (7), we examined whether RPA was inhibitory to 17-3-1-mediated stimulation of DNA synthesis by Polε. Even though RPA is inhibitory to 17-3-1-dependent DNA synthesis by Polε, a considerable stimulation of Polε activity still occurred under the experimental conditions used (Fig. 1*A*, lanes 2–4). The Polε holoenzyme is comprised of the Pol2 catalytic subunit and the Dpb2, Dpb3, and Dpb4 accessory subunits. To determine whether the accessory subunits were required for Polε stimulation by the 17-3-1 clamp and its loader, next we examined the effect of 17-3-1/Rad24-RFC on DNA synthesis by the Pol2 subunit alone. Rather surprisingly, the degree of stimulation conferred by the 17-3-1 clamp on Pol2 activity was nearly the same as that seen with the Polε holoenzyme (Fig. 1*A*, compare lanes 3 and 11), but RPA exerted a more pronounced inhibitory effect on Pol2 than on Polε (Fig. 1*A*, compare lanes 11 and 12 with lanes 3 and 4). To exclude the possibility of any DNA polymerase contamination in purified 17-3-1 clamp or Rad24-RFC, we verified the lack of any DNA synthesis activity in these preparations (Fig. 1*D*, lanes 7–9).

The loading of yeast 17-3-1 clamp onto 5′-recessed DNA junctions occurs most efficiently at ∼100 mM NaCl, and this salt concentration is optimal for activation of Mec1-Ddc2 kinase (11). We thus determined whether the 17-3-1 clamp–dependent activation of Polε exhibits a similar requirement for salt. Even though a significant stimulation of DNA synthesis activity of Polε as well as of Pol2 could be seen in the absence of any salt, maximum primer usage and stimulation occurred at 100 mM (Fig. 1*B*).

### Loading of 17-3-1 clamp at the 3′-recessed DNA junction

Although the inhibitory effect of RPA on 17-3-1-dependent stimulation of synthesis by Polε on circular DNA suggested that the clamp was being loaded at the 3′-recessed junction, the possibility that the clamp was loaded onto the 5′-recessed junction and subsequently diffused to the 3′-junction could not be excluded. Therefore, to ascertain whether the clamp was loaded at the 3′-recessed DNA junction, we examined the stimulation of Polε synthesis by the 17-3-1 clamp using a linear DNA substrate consisting of a 44-mer primer annealed to a 75-mer template, and in which biotin-bound streptavidin was attached at both ends of the template, that not only prevents sliding off of the checkpoint clamp after it had been loaded onto DNA by Rad24-RFC but also limits the loading of 17-3-1 onto the 3′-recessed junction of the DNA substrate (Fig. 1*C*). The stimulatory effect of the clamp on DNA synthesis by Polε, as well as by the Pol2 subunit alone, was observed on the biotin–streptavidin-bound linear DNA; and a marked inhibition of activity occurred when RPA was included in the reaction along with the 17-3-1 clamp and its clamp loader (Fig. 1*C*). These results suggested that similar to the loading of PCNA by RFC, the 17-3-1 clamp is loaded onto the 3′-recessed DNA junction by Rad24-RFC for DNA synthesis by Polε.

### Requirement of Rad24-RFC and ATP for 17-3-1-dependent DNA synthesis by Polε

The observation that the human 9-1-1 clamp can stimulate the activities of proteins such as Polβ, Fen1, and DNA ligase I in the absence of the hRad17-RFC clamp loader on both linear and circular DNA substrates (13, 14, 15, 16) raised the possibility that the observed stimulation of DNA synthesis by Polε by the 17-3-1 clamp may be independent of its loading onto DNA by Rad24-RFC. Studies with 17-3-1 and Rad24-RFC in reactions lacking RPA have shown that similar to the loading of PCNA by RFC, the loading of 17-3-1 clamp at the 3′-recessed junction requires both the Rad24-RFC clamp loader and ATP (3), and similar to the mechanism of release of RFC from PCNA, upon ATP hydrolysis, the 17-3-1 clamp is released from the clamp loader, allowing the clamp to slide along the DNA (1, 3, 17, 18, 19, 20, 21). To ascertain whether 17-3-1 functions as a DNA sliding clamp for Polε in a manner akin to the mechanism of PCNA, we examined the effect of 17-3-1 on DNA synthesis by Polε under conditions that prevent loading of the clamp onto DNA by Rad24-RFC. As shown in Figure 1*D*, Polε activity was not stimulated in reactions that lacked either the Rad24-RFC loader, ATP, or the clamp itself; Polε stimulation occurred only when the 17-3-1 clamp, its loader, and ATP were all present (Fig. 1*D*, lanes 5 and 14). The requirement of Rad24-RFC and ATP for 17-3-1-dependent stimulation of DNA synthesis by Polε conforms with the role of Rad24-RFC in ATP-dependent loading of 17-3-1 at the 3′-recessed DNA junction and in the release of 17-3-1 clamp upon ATP hydrolysis, which then allows the Polε-bound 17-3-1 clamp to slide along DNA.

### The 17-3-1 clamp elevates the catalytic efficiency of correct nucleotide incorporation by Polε

**Figure 2:**
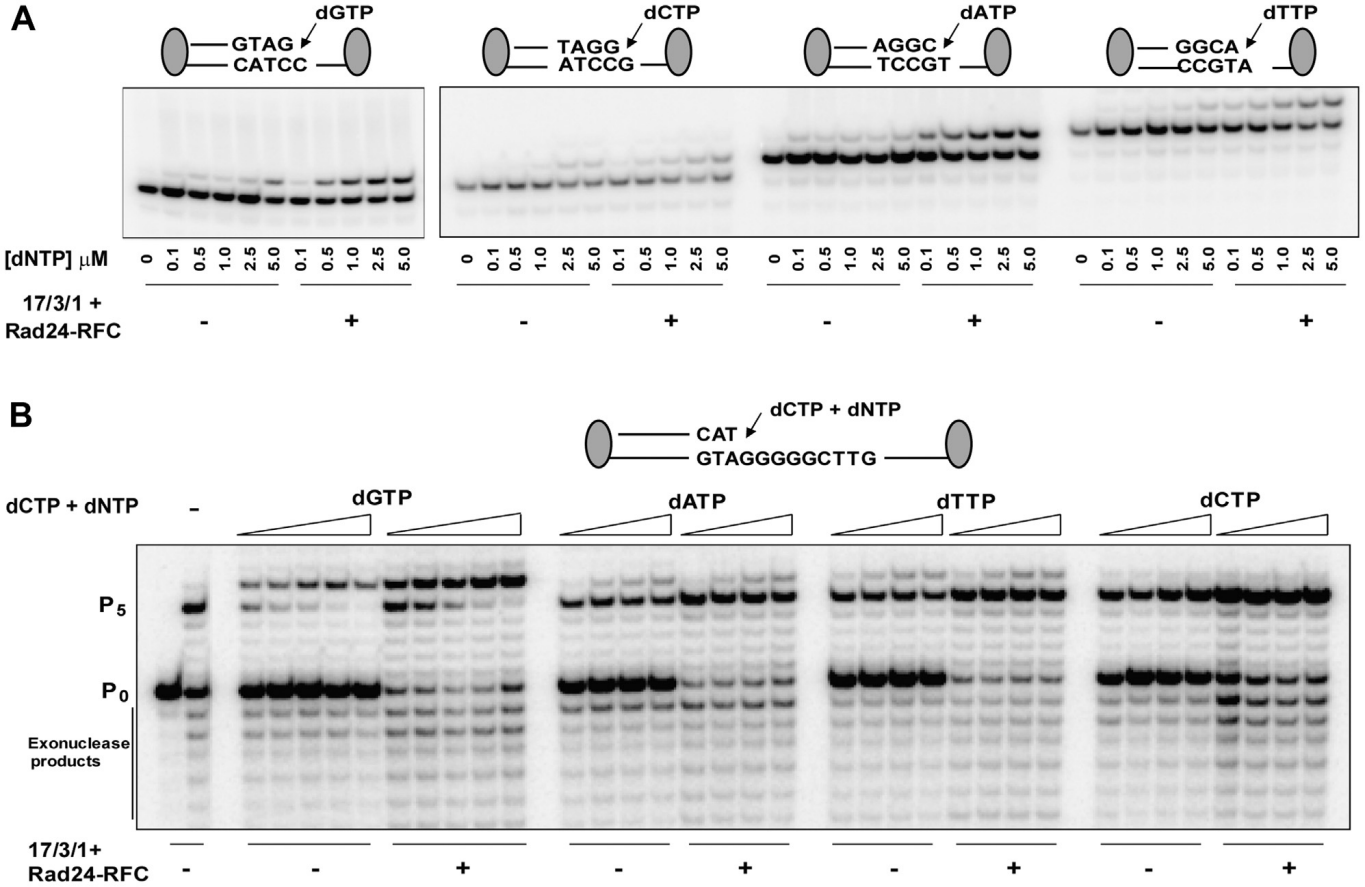
**17-3-1 stimulates the catalytic efficiency of correct nucleotide incorporation by Polε.***A*, incorporation of correct nucleotide opposite different template residues by Pol2 (exo-) in the presence or the absence of 17-3-1 and Rad24-RFC. The primer-template DNA substrate (10 nM) was mixed with Pol2 (0.5 nM) in the absence or the presence of 17-3-1 (40 ng) and Rad24-RFC (10 ng) at 30 °C. The reactions were initiated by adding increasing concentrations of a single deoxynucleotide and further incubated for 2.5 min. Each DNA substrate contained biotin–streptavidin complexes at both ends. *B*, the 17-3-1 clamp stimulates the incorporation of correct nucleotide but not of incorrect nucleotide by Polε. Primer extension reactions were carried out with dCTP (50 μM) on biotin–streptavidin-bound DNA substrate (5 nM), Polε (0.5 nM), and various concentrations of correct or incorrect dNTP (0.1, 0.5, 1, 2.5, and 5 μM for correct dGTP and 100, 200, 500, and 1000 μM for incorrect dNTPs) in the absence or the presence of 17-3-1 (40 ng), and Rad24-RFC (10 ng) for 2.5 min at 30 °C. A running start dCTP (50 μM) control reaction was carried in the absence of any other added dNTP (lane 2). The bands below the P_0_ position of the primer are exonuclease products formed by Polε. Lane 1, DNA substrate alone. Pol2, DNA polymerase 2; Polε, DNA polymerase ε; RFC, replication factor C.

Since 17-3-1 stimulates DNA synthesis by Polε or its Pol2 catalytic subunit to the same extent (Fig. 1*A*), we next determined whether there is a stimulatory effect on the catalytic efficiency of Pol2 for nucleotide incorporation. For these experiments, we carried out steady-state kinetic analyses with the Pol2 catalytic subunit lacking the proofreading 3′→5′ exonuclease activity. As shown in Figure 2*A* and Table 1, overall, the clamp induced an ∼10-fold increase in the catalytic efficiency of correct nucleotide incorporation.

**Table 1:** Stimulation of catalytic efficiency of correct nucleotide incorporation by Pol2 (exo-) catalytic subunit of Polε in the presence of 17-3-1 clamp and Rad24-RFC clamp loader

Template nucleotide	dNTP added	17-3-1 + Rad24-RFC	*k*_cat_ (nM/min)	*K*_*m*_ (μM)	*k*_cat_/*K*_*m*_	Fold difference
C	dGTP	−	2.3 ± 0.7	1.1 ± 0.3	2.1	1
		+	3.3 ± 0.09	0.17 ± 0.04	19.4	9.2
G	dCTP	−	1.2 ± 0.22	1.2 ± 0.45	1.0	1
		+	2.1 ± 0.22	0.15 ± 0.08	14	14
A	dTTP	−	1.8 ± 0.27	0.94 ± 0.33	1.9	1
		+	3.1 ± 0.4	0.21 ± 0.03	15	7.9
T	dATP	−	0.75 ± 0.06	0.83 ± 0.09	0.9	1
		+	2.9 ± 0.13	0.29 ± 0.02	10	11

To determine whether the 17-3-1 clamp increases nucleotide misincorporation by Polε, we examined DNA synthesis on a linear DNA substrate in which the template contained a sequence of 5 G residues followed by a C (Fig. 2*B*). In primer extension reactions, dCTP was added to initiate DNA synthesis opposite the template G residues, and increasing concentrations of either dGTP, dATP, dTTP, or dCTP were added to assess the effects of 17-3-1 clamp on nucleotide misincorporation. As shown in Figure 2*B*, 17-3-1 was highly stimulatory to the incorporation of dGTP opposite template C, and stimulation could be seen even with as little as 0.1 μM dGTP. However, the incorporation of the wrong nucleotides dATP, dTTP, or dCTP opposite template C was not enhanced by the clamp, even when as much as 1000 μM of the dNTP were used. Hence, 17-3-1 stimulates the incorporation of correct but not incorrect nucleotides by Polε.

### A canonical PIP domain in the Pol2 catalytic subunit mediates interaction of Polε with the 17-3-1 clamp

**Figure 3:**
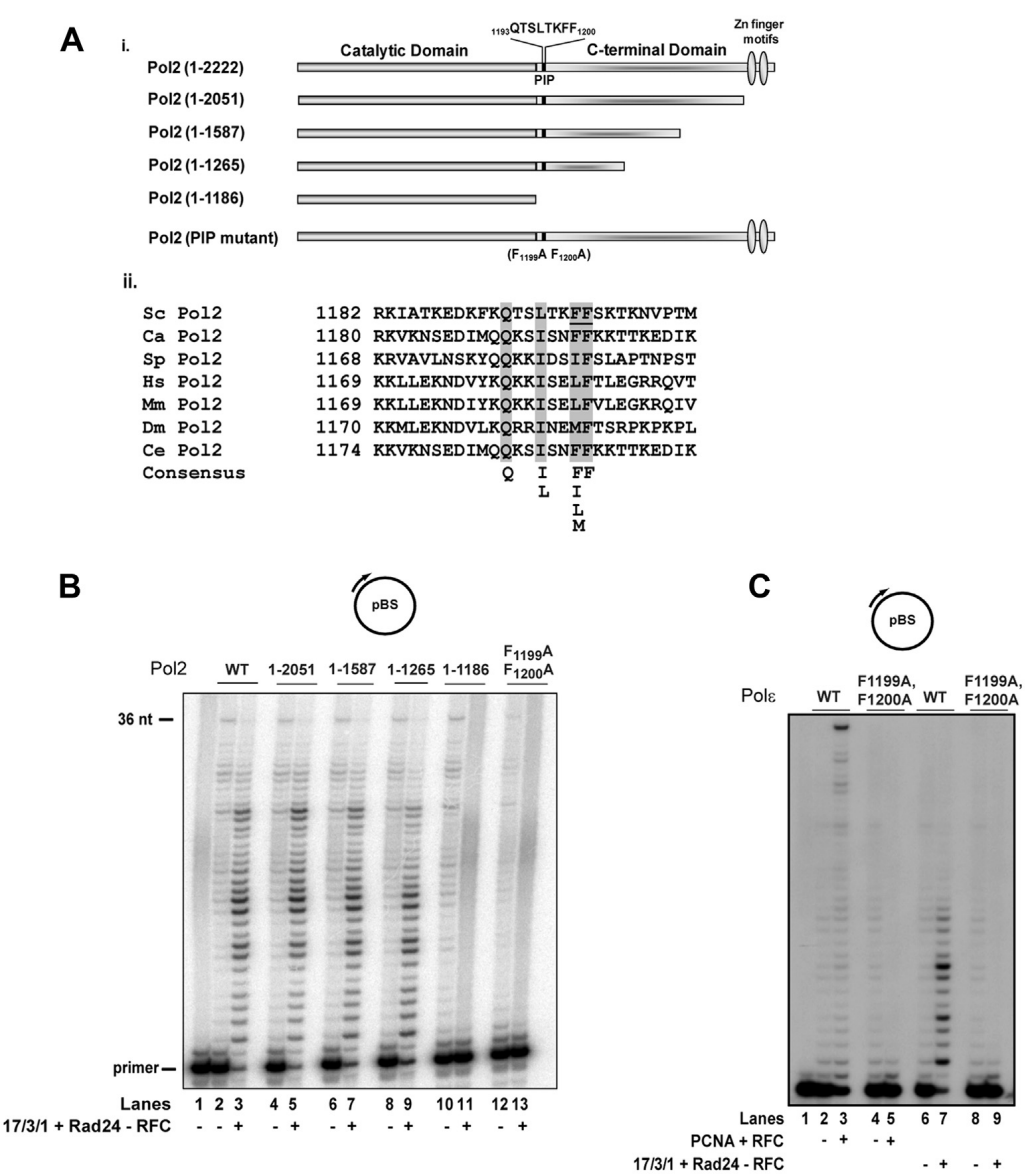
**A single PIP domain in Pol2 mediates binding of Polε to both the 17-3-1 clamp and PCNA.***A*, *i*, schematic representation of C-terminal deletions and of the PIP domain in Pol2. The catalytic and carboxyl terminal domain, the two zinc-finger motifs in the C terminus, and the positions of C-terminal truncations and of the PIP domain in Pol2 are indicated. *ii*, sequence alignment of the conserved PIP motifs in Pol2 from *Saccharomyces cerevisiae*, *Candida albicans*, *Schizosaccharomyces pombe*, *Homo sapiens*, *Mus musculus*, *Drosophila melanogaster*, and *Caenorhabditis elegans*. *B*, effect of C-terminal deletions and of mutations in the PIP domain on 17-3-1-dependent stimulation of DNA synthesis by Pol2. 0.5 nM of Pol2 was incubated with a 5 nM circular ssDNA in the presence of dNTPs under standard reaction conditions. As indicated, the reactions were carried out in the presence or the absence of 17-3-1 (40 ng) and Rad24-RFC (10 ng). Lane 1, DNA substrate alone; lanes 2 and 3, WT Pol2; lanes 4 to 11, Pol2 with different C-terminal truncations; lanes 12 and 13, Pol2 with F1199A, F1200A mutations in the PIP domain. *C*, mutational inactivation of Pol2 PIP renders Polε defective in DNA synthesis with the 17-3-1 clamp as well as with PCNA. 0.5 nM of WT Polε, lanes 2, 3, 6, and 7, or Polε with F1199A, F1200A mutations in Pol2 PIP, lanes 4, 5, 8, and 9, was incubated with the circular ssDNA substrate (5 nM) in the presence of 100 μM dGTP, dCTP, and dATP under standard reaction conditions. As indicated, the reactions were carried out in the presence or the absence of 17-3-1 (40 ng) and Rad24-RFC (10 ng) or PCNA (100 ng) and RFC (25 ng). Lane 1, DNA substrate alone. PCNA, proliferating cell nuclear antigen; PIP, PCNA-interacting peptide; Pol2, DNA polymerase 2; Polε, DNA polymerase ε; RFC, replication factor C.

Many proteins that interact with the interdomain connector loop (IDCL) region of PCNA do so *via* a conserved PIP sequence motif. The region of Pol2 just C terminal to the polymerase domain harbors the sequence QTSLTKFF, between residues 1193 and 1200, which strongly resembles the consensus PIP motif and is highly conserved among Pol2 counterparts in eukaryotes (Fig. 3*A*). To determine whether this sequence or some other region was involved in the binding of 17-3-1 clamp, we made a series of deletions of the Pol2 C terminus (Fig. 3*A*, i) and examined the effect of 17-3-1/Rad24-RFC on DNA synthesis. Whereas DNA synthesis by Pol2 proteins lacking the C terminus beyond residue 1265 could be stimulated in the presence of the 17-3-1 clamp and its loader (Fig. 3*B*, compare lanes 8 and 9), 17-3-1-mediated stimulation of DNA synthesis did not occur with the Pol2 (1–1186) protein (Fig. 3*B*, compare lane 9 with lane 11). These observations suggested that the Pol2 region between residues 1186 and 1265 was required for clamp binding. Since this region contains the conserved PIP motif, we tested whether this motif affects Pol2 binding of the 17-3-1 clamp. For this purpose, the two highly conserved hydrophobic F1199 and F1200 residues in this sequence were changed to alanines, and the mutant Pol2 protein was examined for its response to 17-3-1-mediated activation of synthesis. The lack of stimulation of 17-3-1-dependent DNA synthesis by the F1199A, F1200A Pol2 mutant protein (Fig. 3*B*, lane 13) corroborates a role of this PIP domain in the binding of 17-3-1 clamp.

Since the Polε holoenzyme is comprised of the Pol2, Dpb2, Dpb3, and Dpb4 subunits, the possibility existed that in addition to the functional PIP in the Pol2 subunit, Polε could also bind the 17-3-1 clamp *via* these other subunits. And if the roles of clamp-binding domains in different subunits were redundant, then the stimulation of Polε DNA synthesis by 17-3-1 could still occur in the absence of functional Pol2 PIP. To test for this, WT Polε and the *pip* mutant Polε in which the Pol2 subunit harbors the F1999A, F1200A mutations were examined for DNA synthesis with 17-3-1 and Rad24-RFC. As shown in Figure 3*C*, the 17-3-1-dependent stimulation of DNA synthesis by Polε was inhibited by the *pol2 pip* mutation (compare lanes 7 and 9), indicating that the accessory subunits do not contribute to Polε interaction with 17-3-1.

### Requirement of Pol2 PIP for PCNA binding by Polε

The strong resemblance of the QTSLTKFF sequence to the canonical PCNA-binding PIP motif suggested that this motif could also be involved in PCNA binding by Polε. To verify this, we examined the effect of the F1999A, F1200A mutations on DNA synthesis by Polε in the presence of PCNA and RFC. As shown in Figure 3*C*, PCNA-dependent stimulation of DNA synthesis by Polε was inhibited by the F1999A, F1200A mutations (compare lanes 3 and 5), indicating a role for this PIP motif in PCNA binding also.

## Discussion

Our findings provide the first example where the 17-3-1 clamp, loaded onto 3′-recessed DNA junctions by Rad24-RFC, functions as a sliding clamp for DNA synthesis by Polε. In 17-3-1-dependent DNA synthesis, the catalytic efficiency of Polε for the incorporation of correct nucleotide is elevated. And in contrast to PCNA that exerts an increase in processivity of Polε, DNA synthesis with the 17-3-1 clamp remains distributive. Also noteworthy is the finding that the same single PIP domain within the Pol2 subunit mediates Polε interaction with both the 17-3-1 clamp and PCNA. The crystal structure of human 9-1-1 clamp has indicated that IDCL in each of the 9-1-1 subunits could bind to different partners (22, 23, 24). Thus, Pol2 may bind the 17-3-1 clamp *via* its PIP in a manner similar to that observed for the binding of other PIPs to PCNA, in which the conserved hydrophobic residues of the PIP interact with the hydrophobic residues in the IDCL of PCNA. However, while these hydrophobic interactions between the Pol2 PIP and the IDCL of either PCNA or 17-3-1 would be required for functional interactions with Polε, there must be other structural features in the Pol2 PIP and possibly the surrounding region that allow it to dually bind the 17-3-1 clamp and PCNA. Those features would likely be absent in canonical PIPs, which can only bind PCNA and not the 17-3-1 clamp, as for example, the PIP motifs of the Pol3 or Pol32 subunit of Polδ (25).

A function of 17-3-1-dependent DNA synthesis by Polε in DNA repair processes but not in DNA replication is indicated from the lack of any significant effect of mutational inactivation of either Rad24, the components of 17-3-1 clamp, or the Pol2 PIP on survival of undamaged yeast cells. A role of Polε in DNA synthesis during base excision repair (BER) was identified in studies done with nuclear extracts prepared from yeast cells harboring a temperature-sensitive mutation in the Pol2 subunit of Polε (26). In another study with cell-free yeast extracts, the Pol2 subunit of Polε and the Ddc1 subunit of 17-3-1 clamp were shown to be crosslinked to nicked DNA that was formed in the course of BER (27). Both these studies raise the possibility for a role of 17-3-1-dependent DNA synthesis by Polε during BER and possibly in other DNA repair processes that occur predominantly in the G1 and G2 phases of the cell cycle. 17-3-1-dependent recruitment of Polε could also come into play in S phase when processive replication by Polδ is stalled on the leading strand (28); and DNA synthesis by Polε in conjunction with the 17-3-1 clamp or PCNA may provide alternate means of replication through such stall sites.

## Experimental procedures

### Purification of proteins

Proteins were overexpressed by cloning the individual ORF under control of the GAL-PGK promoter in various plasmids, either natively or in fusion with the glutathione-*S*-transferase (GST) gene. Proteins were expressed in and purified from the protease-deficient yeast strain YRP654 (*MATα ura3-52 trp1Δ leu2Δ1 his3-Δ200 pep4::HIS3 prb1Δ1.6R can1 GAL*) (29). To purify the full-length Pol2 catalytic subunit alone, the Pol2 D290A, E292A (3′→5′) exonuclease-defective protein, or the Pol2 F1199A, F1200A pip mutant protein, GST-tagged proteins were expressed from plasmids pBJ1257, pBJ1296, and pPOL503, respectively. The Pol2 F1199A, F1200A pip and D290A, E292A (3′→5′) exonuclease-defective mutations were generated by the quick change method using mutagenic PCR primers. All mutations were confirmed by sequencing. The C-terminal truncations Pol2(1–2052), Pol2(1–1586), and Pol2(1–1265) were generated by ligation of a SMURFT linker (5′–TTAAGTTAACTTAA-3′) at the restriction endonuclease sites XbaI (+6153 *POL2* ORF), PstI (+4756 *POL2* ORF), or NcoI (+3791 *POL2* ORF), respectively, within the *POL2* gene. GST-tagged Pol2(1–2052), Pol2(1–1586), and Pol2(1–1265) proteins were expressed from plasmids pPOL496, pPOL500, and pPOL495, respectively. The GST-Pol2(1–1187) protein was expressed and purified as described previously (30). To express the WT Polε holoenzyme, or the Polε pip^−^ holoenzyme, yeast cells were cotransformed with either pBJ1257 or pPOL503, respectively, and pJL6, which expresses the Dpb2, Dpb3, and Dpb4 proteins (31). To purify the Pol2 catalytic subunit and its various mutant derivatives, cells were broken in 1× Coomassie brilliant blue containing 500 mM NaCl. Proteins were bound to glutathione-sepharose fast flow, washed with 1× GST binding buffer (GBB) containing 1 M NaCl, followed by equilibration in 1× GBB with 150 mM NaCl. Protein was released from the matrix by overnight incubation with PreScission protease at 4 °C. To purify the Polε holoenzyme, cells were broken in 1× cell breakage buffer containing 250 mM NaCl, and protein was precipitated by the addition of 0.28 g/ml ammonium sulfate. The harvested protein was resuspended in 1× GBB_250_ and dialyzed overnight in 100 volumes of 1× GBB_250_. Polε was purified in a similar manner to the Pol2 subunit, except that after PreScission protease treatment, the complex was further purified by gel filtration on a Superdex PC200 3.2/30 column (GE Lifesciences) in 1× GBB_150_. Yeast Polδ, PCNA, RFC, and RPA were purified as described (25). Yeast Rad17/GST-Mec3/Ddc1 and GST-Rad24-RFC were expressed in yeast strain YRP645 harboring either plasmids pBL760 and pBL764 or plasmids pBL766 and pBL422, respectively (3). The Rad17/Mec3/Ddc1 and Rad24-RFC complexes were purified by glutathione sepharose affinity chromatography followed by elution by PreScission protease treatment under conditions similar to that used for Polε. Each complex was then further purified by gel filtration on Superdex PC200 3.2/30 column (GE Lifesciences) to achieve a homogenous preparation of complexes with equimolar ratio as described (32). Proteins were concentrated using a microcon 30 (Amicon), aliquoted, and frozen at −70 °C freezer.

### DNA polymerase assays

The linear DNA substrates consisted of a 75-nucleotide oligomer template (biotin-5′-AGC AAG TCA CCA ATG TCT AAG AGT TCG TAT TA TGC CTA CAC TGG AGT ACC GGA GCA TCG TCG TGA CTG GGA AAAC-3′-biotin), which contained one biotin molecule at each end annealed to a 5′ ^32^P-labeled primer in a 1.5:1 ratio. The following oligonucleotide primers were used to generate the various DNA substrates indicated in the text: N4264 (5′-GTT TTC CCA GTC ACG ACG ATG CTC CGG TAC TCC AGT GTA G-3′), N4265 (5′-GTT TTC CCA GTC ACG ACG ATG CTC CGG TAC TCC AGT GTA GG-3′), N4266 (5′-GTT TTC CCA GTC ACG ACG ATG CTC CGG TAC TCC AGT GTA GGC-3′), N4267 (5′-GTT TTC CCA GTC ACG ACG ATG CTC CGG TAC TCC AGT GTA GGC A-3′), N4309 (5′-GTT TTC CCA GTC ACG ACG ATG CTC CGG TAC TCC AGT GTA GGC AT-3′), or N4456 (5′-GTT TTC CCA GTC ACG ACG ATG CTC CGG TAC TC-3′). Streptavidin was then bound to the ends of the template DNA by incubation of the linear primer:template substrate with streptavidin at a 1:10 ratio for 15 min at 4 °C, before their addition to the DNA polymerase reactions. The circular DNA substrate consisted of phage DNA from a pBluescript derivative (∼3 kb) annealed to a 5′-P^32^ labeled primer (5′-CCC CCG ATT TAG AGC TTG ACG GGG AAA CCG GCG AAC GTG GC-3′), which hybridizes near the F1 origin of plasmid. The standard DNA polymerase reaction (10 μl) contained 40 mM Tris–HCl, pH 7.5, 5 mM MgCl_2_, 100 mM NaCl, 500 μM ATP, 100 μM of each dNTP, 1 mM DTT, 10% glycerol, 100 μg/ml bovine serum albumin, and 10 nM DNA substrate. DNA synthesis was limited to only 36 nucleotides on circular DNA by omitting dTTP from the reactions. As indicated in the figure legends, reactions in addition contained 100 ng PCNA, 25 ng RFC, 200 ng RPA, 40 ng Rad17/Mec3/Ddc1, and/or 10 ng Rad24-RFC. Assays were assembled on ice and shifted to 30 °C for 2 min before the addition of 0.5 nM DNA polymerase. Reactions were further incubated at 30 °C for 10 min and terminated by the addition of loading buffer (40 μl) containing 20 mM EDTA, 95% formamide, 0.3% bromphenol blue, and 0.3% cyanol blue. Reaction products were resolved on 10% polyacrylamide gels containing 8 M urea and visualized using a STORM PhosphoImager (Molecular Dynamics), and quantification of percentage of primer utilized was done using ImageQuant software (GE Healthcare).

### Fidelity of Polε in the presence of 17-3-1 clamp

Steady-state kinetic analyses for deoxynucleotide incorporation were performed under standard reaction conditions except that only a single dNTP was added to the reaction at various concentrations indicated in the figure legend. Reactions were assembled on ice by mixing 0.5 nM of exonuclease-defective Pol2-4 protein with the various DNA substrates (10 nM) and in the presence or the absence of 17-3-1 and Rad24-RFC. Reactions were shifted to 30 °C for 2 min, and DNA synthesis was initiated by the addition of various concentrations of dNTP. Reactions were further incubated for 2.5 min at 30 °C and terminated by the addition of loading buffer (40 μl) containing 20 mM EDTA, 95% formamide, 0.3% bromphenol blue, and 0.3% cyanol blue. The reaction products were resolved on 10% polyacrylamide gels containing 8 M urea, and gel band intensities of the substrates and products were quantitated by PhosphorImager and the ImageQuant software. The percentage of primer extension was plotted as a function of dNTP concentration, and the data were fit by nonlinear regression using SigmaPlot 5.0 (SPSS, Inc) to the Michaelis–Menten equation describing a hyperbola, *v* = (*k*_cat_[E] × [dNTP])/(*K*_*m*_+ [dNTP]), where [E] refers to enzyme concentration. Apparent *K*_*m*_ and *k*_cat_ steady-state parameters were obtained from the fit and used to calculate the efficiency of deoxynucleotide incorporation (*k*_cat_/*K*_*m*_).

A gel-based assay was used to determine the incorporation of correct and incorrect dNTPs by Polε opposite a template C in a sequence context preceded by 5 G residues. A 75 nucleotide oligonucleotide template DNA containing a run of 5 G residues and harboring 5′ and 3′ biotin moieties (5′-AGC AAG TCA CCA ATG TCT AAG AGT TCG GGG GAT GCC TAC ACT GGA GTA CCG GAG CAT CGT CGT GAC TGG GAA AAC-3′) was annealed to 5′ ^32^P-labeled primer N4309. Reactions were assembled on ice and contained all constituents except DNA Polε. The standard reaction conditions were used except that dCTP was reduced to 50 μM. For correct nucleotide incorporation, dGTP was added at concentrations of 0.1, 0.25, 0.5, 2.5, and 10 μM. Incorrect nucleotides dATP, dTTP, and dCTP were added at concentrations of 100, 500, 1000, and 2000 μM. Where indicated, reactions contained 40 ng Rad17/Mec3/Ddc1 clamp and 10 ng Rad24-RFC. DNA synthesis was initiated by the addition of Polε (0.5 nM) and incubated for 2.5 min at 30 °C. A control reaction was performed containing only 50 μM dCTP and no additional dNTP. Reactions were terminated, and products were resolved on 10% polyacrylamide gels containing 8 M urea. Visualization of the results was done using a Molecular Dynamics STORM PhosphoImager and ImageQuant software.

## Data availability

All the data are contained within the article.

## Conflict of interest

The authors declare that they have no conflicts of interest with the contents of this article.
